# In the Eye of the Beholder: The Impact of Intergenerational Programs from the Perspectives of Their Different Stakeholders

**DOI:** 10.3390/ijerph182211916

**Published:** 2021-11-13

**Authors:** Jiska Cohen-Mansfield

**Affiliations:** 1 Minerva Center for Interdisciplinary Study of End of Life, Tel Aviv University, Tel Aviv 6139001, Israel; jiska@tauex.tau.ac.il; Tel.: +972-3-640-7576; 2 Department of Health Promotion, School of Public Health, Sackler Faculty of Medicine, Tel Aviv University, Tel Aviv 6139001, Israel; 3 Igor Orenstein Chair for the Study of Geriatrics, Tel Aviv University, Tel Aviv 6139001, Israel

**Keywords:** intergenerational programs, stereotyping and bias, social contacts, older adults

## Abstract

Since most evaluations of intergenerational programs (IGPs) focus on the perspective of a single stakeholder group concerning the benefit for themselves, we compared perceptions of multiple stakeholders: older adults, younger adults, and IGP organizers concerning the impact of IGPs on older and young participants. Using a mixed-methods approach, we collected data from thirteen community-based IGPs. The quantitative analyses included a comparison of the different stakeholder groups via ANOVAs and chi-square analyses. In order to identify the reasons for different attribution ratings among stakeholders, we conducted a qualitative analysis of the stakeholders’ comments and responses to open-ended questions using a thematic analysis approach. Overall, participants rated benefits to themselves lower than attributed to them by their counterparts. Differences in ratings may be explained by differences in expectations and needs, cognitive dissonance, as well as a lack of awareness about other participants’ experiences. Given the discrepancies in perception of impact, it is vital to seek input from all stakeholders in order to understand their respective needs and expectations, construct a balanced evaluation, and improve IGP processes and outcomes. Studying a single stakeholder group for project evaluation is likely to provide only one perspective, whereas including all points of view provides a more complete picture.

## 1. Introduction

An aging population that is headed toward outnumbering younger generations raises a range of challenges and requires responses by communities and policymakers [1]. One response is the development of intergenerational programs (IGPs). Among the goals of IGPs are the promotion of intergenerational understanding, alleviation of negative stereotypes [2] and reduction in segregation of older adults (OAs) [3].

Previous studies have reported beneficial outcomes of community- and school-based IGPs for younger persons (YAs), such as improved social skills and acquisition of new knowledge [4,5,6,7,8], improved school performance or academic learning [4,9,10], a decline of negative attitudes towards OAs and aging [8,10,11,12,13,14,15], and the development of friendships with OAs [7,8,10]. However, some studies have also referred to challenges that often stemmed from a lack of preparation or training of YAs prior to participation in intergenerational activities [16].

OAs also reported benefits of community- and school-based IGPs, such as improved physical and cognitive activity [17,18], increased generativity [8,19], increased social activity [20], higher self-esteem or sense of accomplishment [8,15,21,22,23], decreased stress [24], and improved attitudes towards YAs and children [15,22,25,26].

Two studies that reported IGP organizers’ evaluations of IGPs described multiple benefits, including enhancing enjoyment, increasing confidence, improving health, reducing isolation [27], improving attitudes among children towards OAs with dementia, and developing strong bonds and special relationships among participants and parents [28]. Yet, organizers in these studies also reported challenges such as inconsistent attendance, difficulty accommodating some OAs’ special physical and mental conditions, and the need for ongoing training of staff.

Most of the literature examined either OAs’ perspectives [21,26,29,30,31] or YAs’ perspectives [10,12], with few including both [5,7,8,23]. Other studies focused mainly on the perspectives of organizers [27,28]. These studies investigated each group’s perspective on program impact concerning themselves. Other perspectives were explored in the mixed-methods research of Gigliotti, Morris, Smock, Jarrott, and Graham [28], who researched the outlook of organizers and parents of child participants, Skropeta, Colvin, and Sladen [23], who studied childcarers’ perspectives in an IGP playgroup, and a qualitative study by Bullock and Osborne [7], who investigated the perspectives of family members and friends of participants, in addition to those of participants.

To our knowledge, however, no study has examined the perspectives of all three stakeholders—OAs, YAs, and organizers—on the impact of a particular IGP, on themselves, and on the others involved in the program. Studying how each stakeholder group perceives the program’s impact on itself and on other stakeholder groups is crucial for evaluating programs as a whole, and for gaining a fuller understanding of how IGPs affect their participants. Therefore, our study sought to examine (1) how different stakeholders perceive an IGP’s impact on themselves and on others. After finding significant differences among the respective perceptions via quantitative analysis, we further examined (2) the reasons for differences in these perceptions through a qualitative analysis of questionnaire responses.

## 2. Materials and Methods

### 2.1. Participants and Recruitment

We interviewed 84 OAs, 96 YAs, and 21 organizers who were involved with one of 13 IGPs which were designed as community-based experiences for YAs and OAs who met about once a week on average. These IGPs fell into two types based on their content and goals:

Topic-focused IGP type. These programs included a variety of activities focusing on a specific subject: community theater (group 1), intergenerational dancing (2), learning how to play bridge (3), learning photography skills (4), and joint studying of Jewish religious texts (5). Participants in the topic-focused groups included 47 OAs and 32 YAs.

Assistance IGP type. These programs facilitated one-to-one encounters between YAs and OAs, such as YAs visiting OAs’ homes, and providing them with assistance in their daily routines (group 6), a program that specifically sought to promote intergenerational relationships (7), YAs writing OAs’ memoirs (8 and 9), YAs visiting holocaust survivors in their homes (10), Israel Defense Force soldiers visiting an OA at the OA’s home or at a senior club (11), YAs teaching OAs how to use computers and the Internet (12), and YAs assisting OAs with pet care (13). The assistance groups involved 37 OAs and 64 YAs.

### 2.2. Procedures

Data were collected through questionnaires developed on the basis of a literature review and discussions with IGP organizers prior to the initiation of the study. Separate questionnaires were developed for OAs, YAs, and organizers. After obtaining participants’ informed consent, questionnaires were either completed in a personal interview by trained interviewers with an academic degree in the social sciences or were self-administered (completed via an online survey or a printed questionnaire completed by hand). When questions were left unanswered by interviewees, research staff contacted them and followed up on those questions. The in-person interviews were transcribed during the interviews.

### 2.3. Assessments

The questionnaires began with demographic and background questions including age, gender, and education. Subjective health was rated from 1 (poor) to 5 (excellent) based on the global self-rated health item on the SF-36 [32].

The Impact of Intergenerational Programs Questionnaire (IIPQ, based on Cohen-Mansfield and Jensen [33]) asked about the degree and type of benefit (e.g., academic, emotional, social) participants derived from the IGP and the benefits they thought their counterparts derived. Perceived benefits included increased self-esteem, happiness, interest in life, social relationships, and decreased boredom and loneliness for YAs and OAs. Among YAs alone, we evaluated whether perceived benefits also included a decrease in violence and an improvement in their studies. Potential benefits were rated using a 5-point scale: 1—Not at all, 2—To a small extent, 3—To a moderate extent, 4—To a large extent, 5—To a very large extent.

### 2.4. Statistical Approach

We compared the three stakeholder groups (YAs, OAs, and organizers) by displaying means, conducting ANOVAs for ordinal and interval level data, and using percentages and chi-square for nominal level data using IBM® SPSS® Statistics 26. We used ANOVAs to compare the ratings of the respective stakeholder groups concerning the impact of the IGP on various outcome variables separately for YAs and OAs. We used the Benjamini–Hochberg method [34] in order to report the results of a correction for adjustment for multiple comparisons.

In a previous analysis of some of these data [35], we compared this study’s IGP types with respect to various outcome variables reported by participants concerning the impact on themselves. After accounting for multiple comparisons, we did not find significant differences between IGP types in the YAs’ reports concerning the impact on themselves. For OAs there were significant differences in IGP types concerning two (out of six) outcome variables, decrease in boredom and decrease in loneliness. For these outcomes, the assistance type group differed from the topic-focused type group. Therefore, for the outcomes of boredom and loneliness, we compared the reported impact of the informant separately for the assistance type IGPs and the topic-focused type IGPs.

### 2.5. Qualitative Approach

The responses to open-ended questions were analyzed using thematic analysis [36] to elucidate the stakeholders’ perspectives and potential explanations for differing perceptions of benefits. All data were transcribed and coded. In order to assure consistency in coding, several rounds of analysis were undertaken by different members of the research staff. The research staff discussed the different themes and decided on a selection of themes that were most common and relevant to our research questions. Two research staff members then agreed on which quotes to use to illustrate the selected themes. The quotes were translated from Hebrew to English.

## 3. Results

### 3.1. Quantitative Analysis

The background characteristics of the stakeholder groups are described in Table 1. Of the OAs, 82% were female, as compared to 67% for both YAs and organizers. The average age of the OAs was 77, as compared with 23 for YAs. The average age of organizers was 43, and they were more likely to be married with more years of education than the two other stakeholder groups. As reported elsewhere [35], OAs in the assistance type IGPs were less likely to be married in comparison to those in the topic-focused type IPGs, had fewer years of education, and reported worse health status (*p* ≤ 0.01 for all).

As to informants’ ratings of the impact of IGPs on themselves and others, organizers reported a greater positive impact on nearly all measures as compared to the reports of OAs and YAs (see Table 2). When examining the impact on OAs, the ratings of OAs’ and YAs’ ratings of IGP impact were very similar concerning self-esteem and moments of enjoyment. YAs perceived a significantly higher impact than OAs for decrease in boredom and decrease in loneliness. When examining the impact on YAs, the impact was rated significantly higher by OAs than by YAs for self-esteem, moments of enjoyment, and improvement in studies, whereas YAs rated IGP impact on themselves as higher than attributed to them by OAs for adding interest to life, decreasing boredom, and increasing opportunities for social relationships.

Earlier analyses of data from this sample revealed that there were significant differences between topic-focused IGPs and assistance type IGPs in OAs’ self-report of decrease in boredom and decrease in loneliness [35]. Thus, in the current study, we compared the different informants’ ratings of benefits separately for the assistance and topic-focused type IGPs (Table 3). We found the same trends in all cases, i.e., the highest ratings were provided by organizers and the lowest by OAs as to the impact on themselves. The differences in perceptions among stakeholders concerning decrease in boredom for OAs were statistically significant in both the topic-focused and assistance type IGPs. The differences in perceptions among stakeholders as to decrease in loneliness for OAs were statistically significant in the topic-focused IGPs, but not in the assistance type IGPs.

### 3.2. Qualitative Analysis

Our qualitative analysis examined the transcripts of the participants’ responses to open-ended questions in order to clarify the reasons for different attribution ratings among stakeholders.

#### 3.2.1. Organizers: Organizational Difficulties and Cognitive Dissonance

Organizers described their job as difficult, citing the pressures of marketing their program (participant #21, age 63, female, organizer, program: assistance) and matching each YA with an OA quickly. “Filtering is hard work, and you have to focus on this. Otherwise, it’s a nightmare for the OAs and for the coordinators and for the YAs too, and that would be a pity because the project is really good” (#14, 29-F-organizer, assistance).

Some organizers attributed challenges to characteristics of OAs and YAs, or to the matching of OAs and YAs: “Sometimes there are individual difficulties, or the match between the young and old is not good, or the OA does not fully understand what it [an IGP] is [about]” (#64, -27-M-organizer, assistance). Or: “Some young people do not really fit; they do not have much patience. Adults - it’s hard for them to bring strangers [into their homes]” (#69, (unknown age-F-organizer, assistance). Often the organizers have no control over who participates in the program, or they feel they must take anyone who agrees to participate because of shortages of YAs, resulting in mismatches and ongoing tension: “For the young women, the difficulty is poor personal discipline, difficulties in understanding the world of old age […]. We get young women from all kinds of homes and backgrounds, and they do not always have sufficient mental maturity to accept situations of crisis, illness, health crises, losses and death experienced by the OA. The personal guidance [we offer] is very much related to the young women’s willingness to share [their experiences] with the coordinator, and in some cases, I do not know about things that happen” (#21, 63-F-organizer-assistance).

#### 3.2.2. Participants: Lack of Awareness of the Other’s Complex Experience and Expectations

In the quantitative analysis, participants tended to rate benefits to themselves lower than they were rated by their counterparts. For example, in the case of the YAs’ self-assessments, they gave the enjoyment and self-esteem variables the lowest ratings. This might be explained by external stress that YAs experience and which affects their IGP experience. For example, one YA described the time-pressure of IGP participation: “Recently I had the exam period. I know that the [IGP] meetings are supposed to be regular, but there is nothing one can do about it. I work two jobs; now I work in three because I do not have exams” (#133, 24-F-assistance).

Other stressors that YAs reported included feelings of sadness about the OAs’ condition: “It’s difficult to see them [OAs] depressed and to try to improve their mood; it’s also hard to hear difficult stories or to cope with silence” (#48, 20-F-assistance). Other YAs were mindful of the proximity of OAs’ deaths: “I find the issue of death difficult. Two weeks ago, two people from the club passed away-suddenly I realized with what population I work” (#68, 20-F-assistance).

Others felt powerless to handle the OAs’ needs. “Sometimes the OAs talk about things that are too difficult to take in. I have my own difficulties that I need to deal with. But I cannot tell them [I have had] ‘enough’ because I cannot contain it” (# 63, 22-F-assistance). These types of stressors diminished the level of IGP benefit felt by YAs, but they were not reported by OAs and were hardly recognized by organizers.

Understandably, OAs were focused on their own needs: “It’s a help, I need these things… I have no one else to do it… Previously, my children did it, and now they are married and live far away. I do not want to burden them” (#43, 77-F-assistance). The stress expressed by some YAs contrasted with the sense of comfort sometimes expressed by OAs: “I’m not alone; it’s the most important thing” (#142, 83-M-assistance).

YAs and organizers rated the benefits to OAs as to decrease in boredom, opportunity for adding social relationships, and decrease in loneliness higher than OAs did for themselves. For example, some OAs said they felt an activity’s effect was only short-term: “The day she arrives, I’m happy. But only on that day” (#88, 74-F-assistance). OAs complained that some YAs were unreliable and unpunctual: “Some young people are late, and I don’t like it” (#94, 76-M-topic-focused).

Disappointed expectations were also reflected in YAs’ complaints about OAs’ behavior and neediness: “Difficulties exist in facing an OA because it is difficult to set boundaries for them. They want, for example, that I stay on after the meeting or that I will visit them even outside of the activity hours” (#28, 20-F-assistance).

#### 3.2.3. OAs’ Underestimation of Benefits for YAs

In the quantitative findings, there was an exception to the pattern that others’ ratings of benefits tended to be higher than self-ratings. When OAs assessed the benefits to YAs in the areas of decrease in boredom, adding interest to life, and opportunities to enhance social relationships, OAs provided lower ratings than did YAs. This may have been influenced by a perception that YAs are naturally busy. Some OAs expressed awareness of YAs’ multiple commitments and the time pressure upon them: “I understand it’s another age and she’s very busy” (#88, 74-F- assistance), or “You suddenly notice all the difficulties experienced by young people, a lot more is demanded from them than was demanded from us when we were young. The tasks and commitments are much harder for them” (#161, 85-F-topic-focused).

Notwithstanding their misgivings, YAs described how IGPs added to their lives in ways not mentioned by other stakeholders. “The activity contributes to the feeling that I am doing something for other people, especially the older adults. Their loneliness at this age is so painful and I’m glad I have an opportunity to relieve it for a few hours” (#157-21-F-assistance). This sentiment concerning YAs was rarely expressed in the qualitative data from OAs and was not reported by organizers.

Similarly, some YAs found that participation in IGP activities enriched their understanding of the world: “I think I learn a lot from them, whether it’s hearing about things they think, or it’s hearing about things they’ve experienced, about history, understanding about people and emotions… and beyond that, I learned a lot about myself” (#59, 18-F-assistance).

For many YAs, the relationship they developed with the OAs was rewarding in the sense of providing a novel and emotional connection: “I think it’s very interesting to have a relationship like this, between someone [who is] 18 years old and a 60-year-old woman. This is unusual and does not happen much unless it’s a relative. It’s very special” (#51, 18-F-assistance).

## 4. Discussion

Our results revealed three types of discrepancies in perceptions among OAs, YAs and organizers regarding perceptions of benefits to OAs and YAs: (a) organizers tended to provide the most beneficial ratings; (b) in general, the respective participants rated benefits to themselves lower than their counterparts attributed to them; and (c) contrary to the general trend in our results, OAs attributed lower ratings to YAs’ improvement in the variables of decrease in boredom, adding interest to life, and adding social relations than YAs rated for themselves.

In attempting to explain these discrepancies, it may be suggested that organizers presented the most positive image of their programs and their work because they may be the most invested stakeholders. Cognitive dissonance may be at work as well, since organizers may need to feel that their work is valuable in order to justify their involvement in a difficult job for which they often feel undercompensated.

Participants tended to rate benefits to themselves lower than their counterparts did, probably because individuals are more aware than others of the limitations of the benefits for themselves. IGP participants tended to focus on their own perspectives and concerns. For example, while OAs appreciated receiving assistance within an IGP, they were concerned that the program might be terminated and expressed disappointment when the YA could not visit [7]. Our findings also revealed that each stakeholder group focused on its own concerns and did not manifest much awareness of other stakeholder groups’ concerns. Disturbed by OAs’ high expectations, YAs seemed to overlook their own shortcomings regarding punctuality and reliability. While OAs were focused on their own difficulties, they appeared less aware of YAs’ struggles than YAs seemed to be of OAs’ struggles and suffering.

The exception to the overall trend in our results tended to occur when stereotypes obstructed individuals’ appreciation of benefits to others. Specifically, YAs may have overestimated the benefits felt by OAs in the variables of loneliness and decrease in boredom because YAs perceived OAs as dependent, lonely, and bored [37,38]. Conversely, OAs perceived YAs as busy with no need for improvement in the realms of decrease in boredom, adding interest to life, or adding social relations, a stereotype that studies have discredited [39,40,41]. The reports of our study participants suggest that the sense of meaningfulness that YAs gain from helping OAs is a benefit of which OAs seem unaware.

The hurdle to mutual understanding between younger and older IGP participants could potentially be mitigated through better matching of OAs and YAs and better provision of guidance and training for them. Our data indicated that even organizers are stressed and constrained in their ability to improve intergenerational understanding due to a lack of funding and resources, as was also described by Ayala et al. (2007) [27], and insufficient training of staff [28]. Encouragement of discussion between OAs and YAs of their respective joys and difficulties may help mutual understanding, though this activity may work better in some programs than in others and may depend on the prescribed goals of the IGP.

A strength of this study is the sample size, which is larger than that of most studies of IGPs. The use of multiple programs was not only necessary to obtain the sample size but likely enhanced generalization. As all of the IGPs were in Israel, more research is necessary to achieve greater generalization to other contexts.

Our research demonstrates not only how different stakeholders in IGPs perceive the benefits and challenges of the programs differently, but also how they attribute different levels of program impact to their fellow stakeholders. The value of such results is especially significant because the vast majority of IGP evaluations utilize only one point of view. It is vital to seek input from all stakeholder groups whenever possible in order to achieve broader and more telling insights into IGPs’ impact.

## 5. Conclusions

This study demonstrates the importance of seeking input from all stakeholders in order to understand their respective needs and expectations, construct a balanced evaluation, and improve the processes and outcomes of IGPs. Using a single stakeholder group for project evaluation provides only one perspective, whereas including diverse groups provides a more complete picture. For this reason, and considering the differences in benefits perceived by various stakeholders, it seems that self-report by one stakeholder provides an incomplete picture. Studying the gaps in perceptions is useful for improving our understanding of each stakeholder group, and helping OAs, YAs and organizers understand each other. This point is best demonstrated by OAs’ significant underestimation of the contribution of IGPs to decreasing YAs’ boredom and loneliness, reinforcing the idea that the study of discrepancies among perspectives is an effective tool for evaluating and improving IGPs.

## Figures and Tables

**Table 1:** Comparison of background variables of the different informants.

	Older Adults [OAs]	Young Adults [YAs]	Organizers	Comparison between 3 Stakeholder Groups	*p*
N	84	96	21	201	
Sex % female	82.14%	66.70%	66.70%	X^2^_2_ = 5.96	0.051
Age M	77.24 ^a^	23.49 ^b^	42.90 ^c^	F_2,194_ = 957.28	<0.001
Marital status married (% married)	30.12%	5.32%	55.00%	X^2^_6_ = 162.57	<0.001
Years of education M	13.25 ^a^	13.43	17.03 ^c^	F_2,170_ = 6.97	0.001
Health status Scale 1–5 ^1^	3.10	No such question	No such question		
Religiosity Scale 1–3 M ^2^	1.59	1.56	1.42	F_2,183_ = 0.45	0.641

^1^ Scale: 1—Poor, 2—Not so good, 3—Quite good, 4—Good, 5—Excellent. ^2^ Scale: 1—Non-religious, 2—Traditional, 3—Religious and orthodox. Significant Scheffe post hoc difference: ^a^ OAs vs. organizers, ^b^ YAs vs. OAs, ^c^ organizers vs. YAs.

**Table 2:** Comparison of outcomes across informants.

Informant Type of Impact	Older Adults [OAs] *n* = 84	Young Adults [YAs] *n* = 96	Organizers *n* = 21	Comparison between 3 Stakeholder Groups	*p*
**Impact on OAs** scale 1–5 ^1^	**Means**				
Self-esteem	2.74 ^a^	2.70	3.85 ^c^	F_2,182_ = 6.18	0.003 *
Moments of enjoyment	3.61 ^a^	3.63	4.40 ^c^	F_2,187_ = 4.66	0.011 *
Decrease in boredom	3.12 ^a^	3.90 ^b^	4.40	F_2,187_ = 13.88	<0.001 *
Adding interest to life	3.86	3.74	4.35 ^c^	F_2,186_ = 3.31	0.039
Opportunity for adding social relationships	2.90 ^a^	3.30	4.20 ^c^	F_2,185_ = 8.68	<0.001 *
Decrease in loneliness	2.96 ^a^	3.79 ^b^	4.20	F_2,184_ = 12.19	<0.001 *
**Impact on YAs** scale 1–5 ^1^					
Self-esteem	3.37	2.24 ^b^	3.10 ^c^	F_2,166_ = 14.68	<0.001 *
Moments of enjoyment	3.75	2.91 ^b^	3.19	F_2,169_ = 10.43	<0.001 *
Decrease in boredom	1.73 ^a^	2.49 ^b^	2.67	F_2,197_ = 7.89	0.001 *
Adding interest to life	2.49 ^a^	3.27 ^b^	3.95	F_2,1__96_ = 11.07	<0.001 *
Opportunity for adding social relationships	2.19	2.78 ^b^	3.05	F_2,195_ = 4.67	0.010 *
Decrease in loneliness	1.40 ^a^	1.86	2.86 ^c^	F_2,197_ = 9.35	<0.001 *
Decrease in violence	1.10 ^a^	1.24	2.05 ^c^	F_2,195_ = 6.07	0.003 *
Improvement in studies	2.64	1.79 ^b^	2.56	F_2,155_ = 7.44	0.001 *

^1^ Scale: 1—Not at all, 2—To a small extent, 3—To a moderate extent, 4—To a large extent, 5—To a very large extent. * Indicates comparisons for which the difference is statistically significant after applying the Benjamini–Hochberg method [34] for correction for multiple comparisons. Significant Scheffe post hoc difference: ^a^ OAs vs. organizers, ^b^ YAs vs. OAs, ^c^ organizers vs. YAs.

**Table 3:** Differences between informant reports of outcomes.

Informant Program	Organizers	Older Adults [OAs]	Young Adults [YAs]	ANOVA F_df_	*p*	N
	Means					
**For Decrease in Boredom for OAs ^1^**						
Topic-focused group	4.50 ^a^	2.76 ^b^	3.86	F_2,73_ = 8.37	0.001	76
Assistance	4.36 ^a^	3.53	3.92	F_2,111_ = 3.41	0.037	114
**For Decrease in Loneliness for OAs ^1^**						
Topic-focused group	4.00 ^a^	2.50 ^b^	3.66	F_2,74_ = 7.76	0.001	77
Assistance	4.29	3.53	3.85	F_2,107_ = 2.27	0.109	110

^1^ Scale: 1—Not at all, 2—To a small extent, 3—To a moderate extent, 4—To a large extent, 5—To a very large extent. Significant Scheffe post hoc difference: ^a^ organizers vs. OAs, ^b^ OAs vs. YAs.

## Data Availability

Qualitative data are not available due to confidentiality concerns. Quantitative data may be available upon request directed to the author, contingent upon approval from the IRB of Tel Aviv University.
